# Proliferating trichilemmal tumor^⋆^

**DOI:** 10.1016/j.abd.2021.11.015

**Published:** 2023-08-03

**Authors:** Andrea Abê Pereira, Jéssica Lüders Bueno, Ana Letícia Boff, Paulo Ricardo Martins Souza

**Affiliations:** a Department of Dermatology, Universidade Federal de Ciências da Saúde de Porto Alegre, Porto Alegre, RS, Brazil; b Department of Dermatology, Santa Casa de Misericórdia de Porto Alegre, Porto Alegre, RS, Brazil

Dear Editor,

Proliferating trichilemmal tumor (PTT) is a rare adnexal neoplasm^1^, ^2^ derived from the outer sheath of the hair follicle and may originate from a pre-existing pilar/trichilemmal cyst as a result of trauma and/or inflammation in the latter.^1^, ^3^ It was first reported in 1966 by Wilson Jones^2^, ^4^, ^5^ and it is benign in most cases^3^, ^4^ but eventually it can be malignant, recur locally, invade adjacent tissues and cause distant metastases.^1^, ^3^, ^4^, ^5^ It often presents as a solitary, soft nodular lesion, measuring 1 to 10 cm in diameter, on the scalp of elderly women.^1^, ^3^ and may be associated with an area of alopecia. More rarely, it can affect other topographies such as the neck, trunk, groin, mons pubis, vulva, gluteal region, and skull base.^1^, ^3^

This report describes the case of a 52-year-old male patient, with no comorbidities, who sought medical care complaining of a lesion on the scalp, with discomfort on palpation, that had been growing for about a year. He had a personal history of scalp cyst removal years before. On clinical examination, a tumor-like, erythematous lesion with a pedunculated structure in the center, measuring approximately 3 cm in its largest diameter, was observed in the parieto-occipital region (Fig. 1). The main hypothesis was trichilemmal cyst with atypical clinical presentation and, for this reason, an incisional spindle biopsy was performed, measuring about 2 cm in its largest diameter for anatomopathological analysis. Histopathology showed a dermal tumor, with no connection with the epidermis, consisting of an expansive lobular proliferation of squamous cells with ample eosinophilic cytoplasm and abrupt keratinization, without the formation of a granular layer - trichilemmal-type keratinization ‒ in the central portion of the lobes (Fig. 2). Some of these cells had atypical nuclei, but no mitosis (Fig. 3), findings consistent with a proliferating trichilemmal tumor. The patient was referred for complete excision of the lesion and showed good evolution, with no signs of recurrence to date.

**Figure 1:**
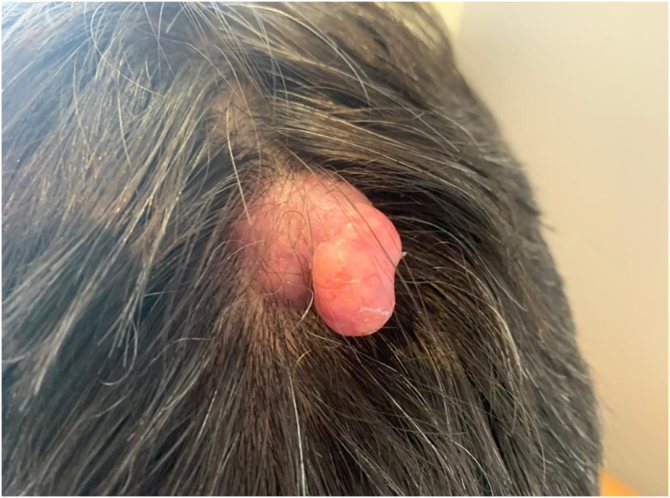
Tumor in the parieto-occipital region

**Figure 2:**
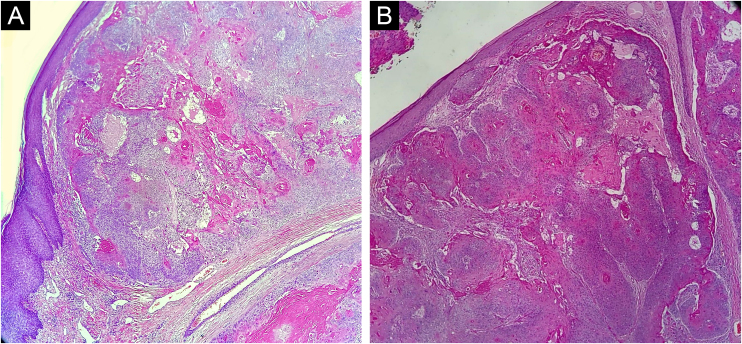
(A and B) Proliferation of lobular squamous tumor islands in the dermis, without connection with the epidermis. There is trichilemmal-type keratinization, consisting of compact and eosinophilic keratin in the center, in two distinct areas (Hematoxylin & eosin, ×40)

**Figure 3:**
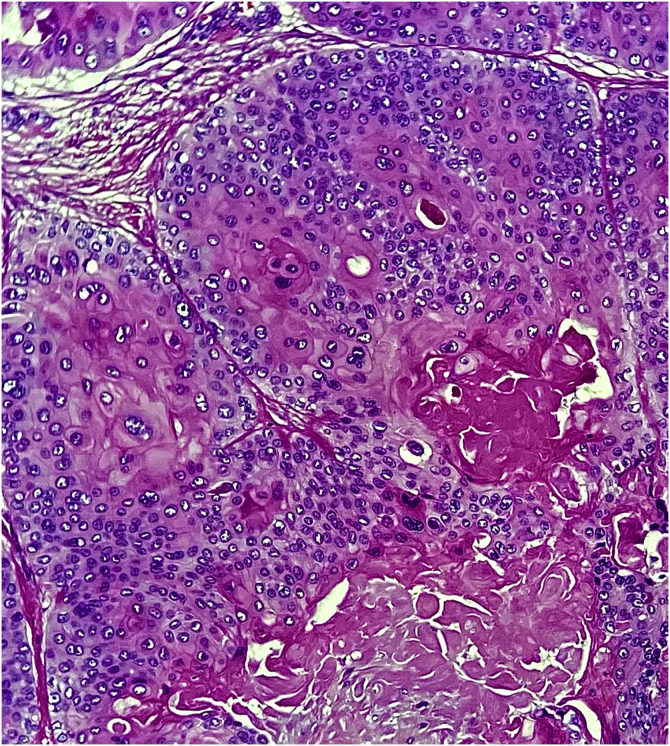
Squamous cells with atypical nuclei, but without mitosis. Towards the central part of the tumor island there is abrupt keratinization of the trichilemmal type (Hematoxylin & eosin, ×400)

The anatomopathological analysis is crucial for the diagnosis of PTT, because clinically, as in the case reported herein, it can be mistaken for a trichilemmal cyst, or other adnexal tumors.^1^ What histopathologically characterizes this tumor is the proliferation of squamous cells with trichilemmal keratinization (defined by the abrupt transition from nucleated to anucleated epithelial cells, with the absence of a granular layer) and varying degrees of atypia.^1^, ^2^, ^3^ The presence of poorly-defined borders, high-grade atypia, aneuploidy, necrosis, cellular pleomorphism and atypical mitoses are related to malignancy (malignant proliferating trichilemmal tumor) and, in this case, distant metastases may rarely appear, even years after the excision of the primary tumor.^1^, ^3^, ^5^ Immunohistochemistry can be used to help in the detection of malignancy.^1^, ^2^

Treatment consists of surgical excision, with margins ranging from 1 cm to large resections, depending on the histopathology of the tumor.^1^, ^3^ Radiotherapy and chemotherapy have been described as alternative or adjuvant treatments.^1^, ^3^ To reduce the risk of recurrence, the evaluation of the margins with Mohs micrographic surgery is the procedure of choice,^1^, ^3^, ^4^ as well as the close follow-up of these patients.^1^, ^3^

## Financial support

None declared.

## Authors' contributions

Andrea Abê Pereira: Critical review of the literature; drafting and editing of the manuscript; approval of the final version of the manuscript; design and planning of the study.

Jéssica Lüders Bueno: Critical review of the literature; approval of the final version of the manuscript; design and planning of the study; drafting and editing of the manuscript.

Ana Letícia Boff: Drafting and editing of the manuscript; collection, analysis and interpretation of data; critical review of the manuscript.

Paulo Ricardo Martins Souza: Approval of the final version of the manuscript; design and planning of the study; drafting and editing of the manuscript; collection, analysis and interpretation of data; intellectual participation in the propaedeutic and/or therapeutic conduct of the studied cases; critical review of the literature; critical review of the manuscript.

## Conflicts of interest

None declared.
